# Evaluation of lateral epicondylopathy, posterior interosseous nerve compression, and plica syndrome as co-existing causes of chronic tennis elbow

**DOI:** 10.1007/s00264-023-05805-x

**Published:** 2023-04-18

**Authors:** Michał Bonczar, Patryk Ostrowski, Martyna Dziedzic, Marcin Kasprzyk, Rafał Obuchowicz, Tomasz Zacharias, Jakub Marchewka, Jerzy Walocha, Mateusz Koziej

**Affiliations:** 1 Department of Anatomy, Jagiellonian University Medical College, Mikołaja Kopernika 12, 33-332, Kraków, Poland; 2 Youthoria, Youth Research Organization, Kraków, Poland; 3 Intermed Medical Center, Zabierzów, Poland; 4 Department of Diagnostic Imaging, Jagiellonian University Medical College, Kraków, Poland; 5 Medic House Medical Center, Kraków, Poland; 6 5th Military Hospital With Polyclinic, Kraków, Poland

**Keywords:** Tennis elbow, Posterior interosseous nerve, Synovial plica, Elbow, Surgery, Diagnosis

## Abstract

**Purpose:**

A great number of patients that suffer from lateral epicondylitis, commonly called tennis elbow (TE), are not successfully treated, meaning, not getting adequate therapeutic effects and the main origin of the pain not being handled appropriately. The hypothesis of the present study is that the inefficiency of the treatment of the chronic TE may often be due to underdiagnosis of posterior interosseous nerve (PIN) entrapment or and plica syndrome, as the authors believe that those pathologies can often occur simultaneously.

**Methods:**

A prospective cross sectional study was conducted. A total of 31 patients met the required criteria.

**Results:**

Thirteen (40.7%) of the patients had more than one source of the lateral elbow pain. Five patients (15.6%) had all three examined pathologies. Six patients (18.8%) had TE and PIN syndrome. Two patients (6.3%) had TE and plica syndrome.

**Conclusion:**

The present study demonstrated concomitant potential sources of lateral elbow pain in patients diagnosed with chronic TE. Our analysis shows how important it is to systematically diagnose patients that present with lateral elbow pain. The clinical characteristics of the three most common causes of chronic lateral elbow pain, meaning, TE, PIN compression, and plicae syndrome were also analyzed. Having adequate knowledge about the clinical aspects of these pathologies can help with a more effective differentiation of the etiology of chronic lateral elbow pain, and with that, a more efficient and cost-effective treatment plan.

## Introduction

**Fig. 1:**
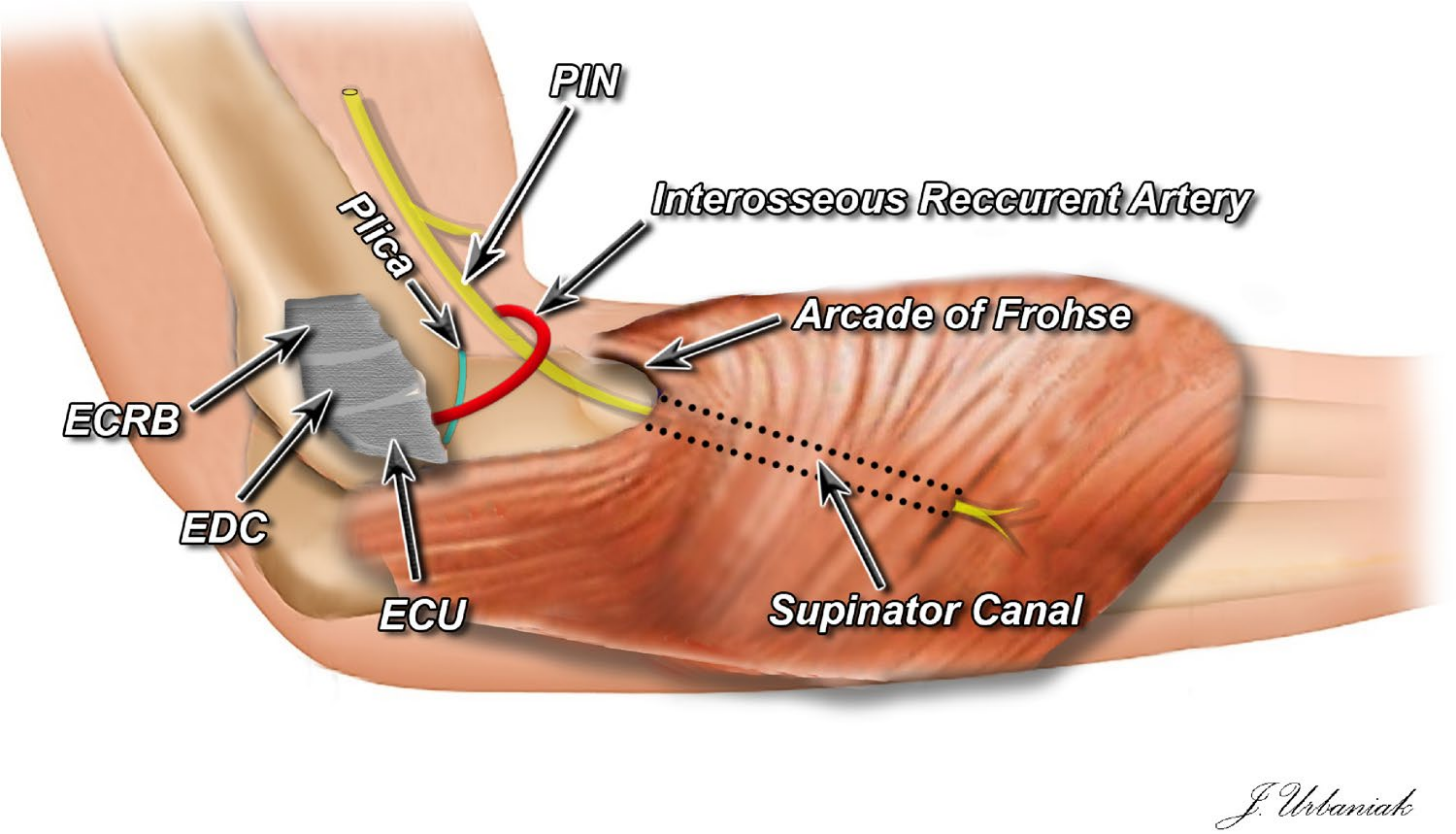
Graphical depiction of the anatomy of the examined sources of the lateral elbow pain. ECRB—extensor carpi radialis brevis. EDC — extensor digitorum communis. ECU — extensor carpi ulnaris. PIN — posterior interosseous nerve. Plica — synovial lateral plica of the elbow

Lateral epicondylitis, commonly called tennis elbow (TE), is a disorder that is often encountered in clinical practice and is estimated to afflict 1 to 3% of individuals annually [1–3]. TE primarily occurs in the recreational tennis player [4, 5]. A great number of patients that suffer from lateral epicondylitis are not successfully treated, meaning, not getting adequate therapeutic effects and the main origin of the pain not being handled appropriately [3]. Lateral elbow pain can have many etiologies. The main pathologies that contribute to lateral elbow pain are said to be TE, posterior interosseous nerve (PIN) entrapment, and plicae syndrome (Fig. 1) [6].

TE is characterized by tenderness at the common extensor origin, which is often exacerbated by wrist extension. It is the most frequent type of myotendinosis but also the most commonly diagnosed elbow pathologic condition [7, 8]. Other possible symptoms/findings associated with TE may be oedema, tendinosis, rupture of the extensors attached to the lateral epicondyle, and uneven bone attachment area, amongst others.

PIN entrapment may be confused with TE because the clinical symptoms, as well as the location and severity of the pain, are often alike [6]. The most frequent compression site of the PIN is the arcade of Frohse, which is a fibrous arch formed by the proximal edge of the superficial head of the supinator muscle [9].

Another pathology that may mimic the clinical symptoms of the TE is plica syndrome. The synovial plicae in itself is a controversial structure, with quite scarce literature concerning its morphological aspects, and diagnostic importance. Its presentation and clinical significance may easily be either overestimated or underestimated, mainly due to no clinical consensus being made for the diagnosis of its pathological form [10]. However, plica syndrome may present with edema, fibrous changes, and cystic remodeling, amongst others.

The hypothesis of the present study is that the inefficiency of the treatment of chronic TE may often be due to the underdiagnosis of PIN entrapment or and plica syndrome, as the authors believe that those pathologies can often occur simultaneously. It must be noted that patients with a diagnosis of chronic TE might suffer from not only inflammation of the common extensor tendons, but also additional sources of lateral elbow pain. This can therefore lead to an incomplete diagnosis, and, subsequently, unsuccessful treatment. In those cases in which the etiology of the pain comes from numerous causes, the differentiation between these different possible aetiologies of the said pain may be extremely difficult, especially when one has to differentiate between TE, PIN compression, and plica syndrome. Therefore, the goal of the present study was to analyze the co-occurrence of these clinically similar pathologies in 31 patients previously diagnosed with the chronic TE. Having adequate knowledge about the clinical aspects and awareness of the frequency of co-occurrence of these pathologies can help with a more effective differentiation of the etiology of chronic lateral elbow pain, and with that, a more efficient and cost-effective treatment plan.

## Patients and methods

### Study group

A prospective cross sectional study was conducted to establish the co-occurrence of the TE, PIN syndrome, and plica syndrome in patients with the previous diagnosis of chronic TE. For this purpose, 50 consecutive patients with a diagnosed chronic TE were invited to participate in the study. The examinations of the patients were conducted between September 2021 and December 2022 in Intermed Medical Center, Kraków, Poland.

The inclusion criteria were set as follows: (1) unilateral chronic TE (lasting as least 6 months), previously diagnosed and confirmed by a medical records; (2) lateral elbow pain; however, only in the upper extremity with the chronic TE; (3) minimal age of 18 years; (4) declaration in writing of the informed consent to participate in the study and for the publication of the results; (5) no contraindications for the ultrasound (US) examination; (6) no history of nervous system diseases; (7) no history of connective tissue diseases; (8) no significant deformities (e.g., amputations) in both upper extremities.

The exclusion criteria were set as follows: (1) failure to meet any of the inclusion conditions; (2) accompanying neurological or psychiatric disorders or ongoing neurological or psychiatric treatment; (3) pregnancy; (4) patients with a history of trauma to the lateral elbow that may interfere the results; (5) inability to fully visualize and/ or measurement of all of the examined structures; (6) significant artifacts that prevented accurate and precise imaging and/ or measurement of all of the examined structures.

Out of the initially gathered group, 19 patients were excluded from the study. Finally, a total of 31 patients met the required criteria.

### Examination and measurements

Detailed medical history regarding the upper extremity was collected from all patients. Subsequently, each patient has been physically examined, including the US assessment. Each examination was performed by three independent researchers, of which two are specialists in orthopaedics and/or radiology and deal with the upper extremity in the daily clinical practice. However, none of the measurements results of the researchers could not differ from one another by more than 0.05 mm. Any discrepancies in the examination identified by the reviewers were resolved by consensus or by the fourth reviewer, also an orthopaedic specialist.

A detailed interview was conducted with each patient. Furthermore, a physical examination has been performed, and on those bases, a preliminary diagnosis was set. Afterwards, each patient was asked to fill two, subjective, pain-related evaluation forms: (1) Patient-Related Tennis Elbow Evaluation form, validated Polish version (PRTEE) [11], and (2) the Disabilities of the Arm, Shoulder and Hand evaluation form, the Quick, validated Polish version (Quick Dash) [12]. Subsequently, an US examination was carried out [Linear Array Probe; 6–12 MHz SP GE; Voluson 730 Expert]. A set of parameters was evaluated in both of patients’ upper extremities.

The parameters regarding the TE: (1) presence of the oedema; (2) presence of the inflammation assessed via a Doppler analysis; (3) presence of the tendinosis; (4) presence of the uneven bone attachment area; (5) presence of the rupture of the attachments of the extensor carpi radialis brevis (ECRB); and/or extensor digitorum communis (EDC) and/or extensor carpi ulnaris (ECU) and/or extensor digiti minimi (EDM).

The parameters regarding the PIN: (1) diameter of the PIN before the supinator canal [mm]; (2) diameter of the PIN in the supinator canal [mm]; (3) diameter of the PIN after the supinator canal [mm]; (4) modeling of the PIN by the arcade of Frohse in a static examination; (5) modeling of the PIN by the arcade of Frohse in a dynamic examination; (6) crossing of the PIN with the recurrent interosseous artery.

The parameters regarding the plica: (1) plicas’ maximal thickness [mm]; (2) presence of the oedema; (3) presence of the fibrous changes; (4) presence of the cystic remodeling; (5) presence of the vascularity in Doppler examination; (6) modeling of the plica by bones.

The US examinations were performed according to the recommendations by Obuchowicz and Bonczar [6].

### Statistical analysis

Statistical analysis was performed with STATISTICA v13.1 (StatSoft Inc., Tulsa, OK, USA). The frequency and percentages presented qualitative features. The Shapiro–Wilk test was used to assess the normal distribution. Quantitative characteristics were presented by means and standard deviation (SD), as well as by simple percentages. Statistical significance was defined as *p* ≤ 0.05. The qualitative variables were compared using the *χ*^2^ test of proportions for categorical variables. Wilcoxon signed-rank test was used to establish potential differences between groups. Spearman’s rank correlation coefficient was used to determine possible correlations between the parameters. The statistical analysis has been checked with the CHAMP checklist [13].

## Results

### Patients’ characteristics

Eventually, thirty-one patients were examined during this study. Patients were between 33 and 68 years of age, with a mean age of 47.09 (SD 8.48). There were 12 (38.71%) women and 19 (61.29%) men who participated in this research. The mean number of examined sources of the lateral elbow pain (TE, PIN syndrome or plica syndrome) was set to be 1.56 (SD 0.76). The mean PRTEE score was found to be 50.33 (SD 19.69), whilst the mean QuickDASH score was set to be 52.02 (SD 18.22).

### Coexistence of the sources of the lateral elbow pain

**Fig. 2:**
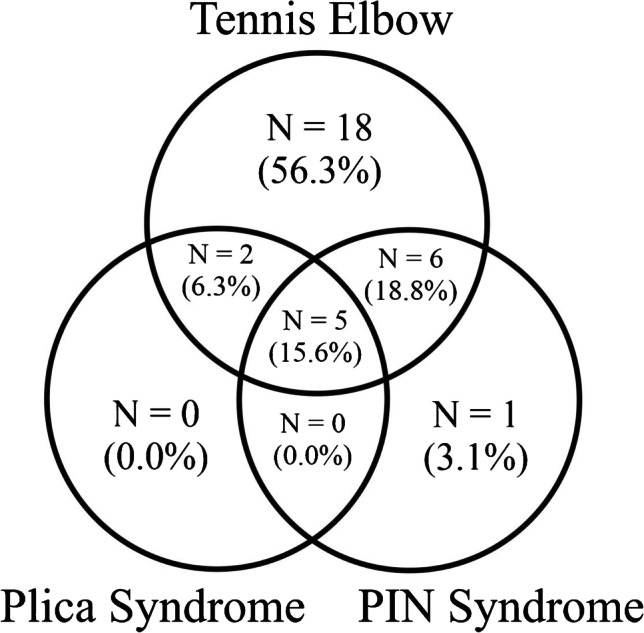
Illustration of the coexistence of the sources of the lateral elbow pain based on the results of the present study

Thirteen (40.7%) of the patients had more than one source of the lateral elbow pain. Five patients (15.6%) had all three examined pathologies. Six patients (18.8%) had TE and PIN syndrome. Two patients (6.3%) had TE and plica syndrome. Figure 2 illustrates the coexistence of the sources of the lateral elbow pain.

### Tennis elbow

**Fig. 3:**
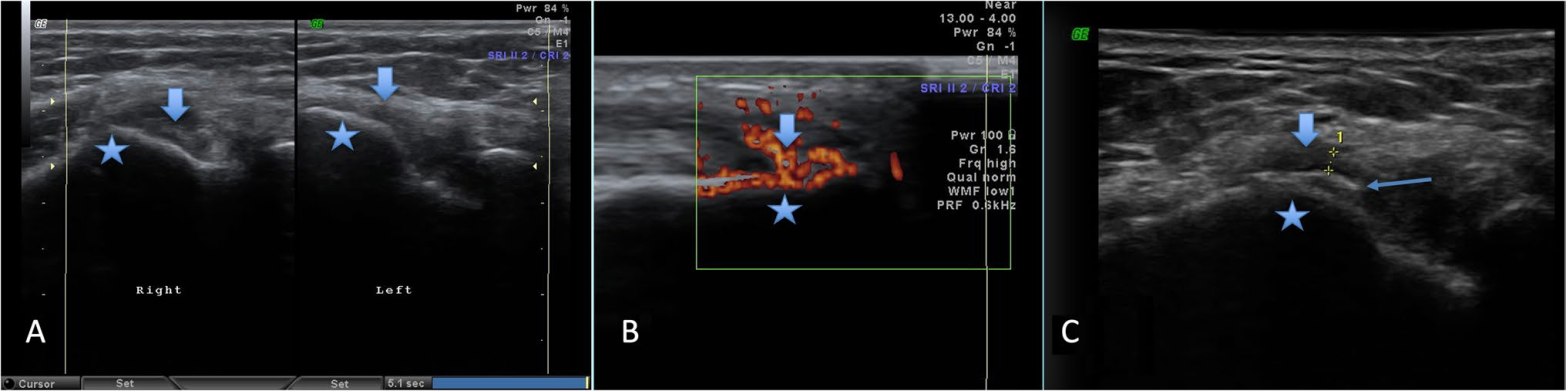
Presents ultrasonographic images of different morbidities of lateral common extensor tendon in the enthesis area. **a** Comparison between tendinosis driven enlargement of irregular tendon (described as right) in comparison with tendon of markedly decreased width (marked as left) what is marker for the tear of the tendon. Star indicates lateral epicondyle of humerus. Arrow indicates common extensor tendons. **b** Power Doppler study of inflamed tendon with color coding of the blood flow in the arterioles developed due to inflammatory angiogenesis. Note high density of the vessels what suggests intense inflammatory process. Star indicates lateral epicondyle of humerus. Arrow indicates common extensor tendons. **c** Degeneration of bone with “double periosteum” sign due to formation of the lytic zone in course of prolonged inflammatory degeneration. Above markedly thinned tendon (partially ruptured) is pointed by calipers. Star indicates lateral epicondyle of humerus. Arrow indicates common extensor tendons. Thin arrow indicates periosteum at the tendon enthesis area

The edema occurred in 25 (80.6%) of the symptomatic limbs, whilst only in four (12.9%) of the asymptomatic limbs. The inflammation occurred in 21 (67.7%) of the symptomatic limbs, whilst only in one (3.2%) of the asymptomatic limbs. The tendinosis occurred in 24 (77.4%) of the symptomatic limbs. For detailed results of the TE examination, regarding all the assessed parameters, please see Table 1. For US images of examined TE, please see Fig. 3.

**Table 1:** Results of the occurrence of the parameters taken for consideration in evaluation of the tennis elbow regarding both research and control samples

Category	Patients with tennis elbow		
	In symptomatic limbs	In asymptomatic limbs	*p* value
*N*	31	31	-
Oedema	25 (80.6%)	4 (12.9%)	0.00
Inflammation	21 (67.7%)	1 (3.2%)	0.00
Tendinosis	24 (77.4%)	4 (12.9%)	0.00
Uneven bone attachment area	17 (54.8%)	3 (9.7%)	0.00
Rupture	1 (3.2%)	0 (0.0%)	0.32

### Posterior interosseous nerve syndrome

**Fig. 4:**
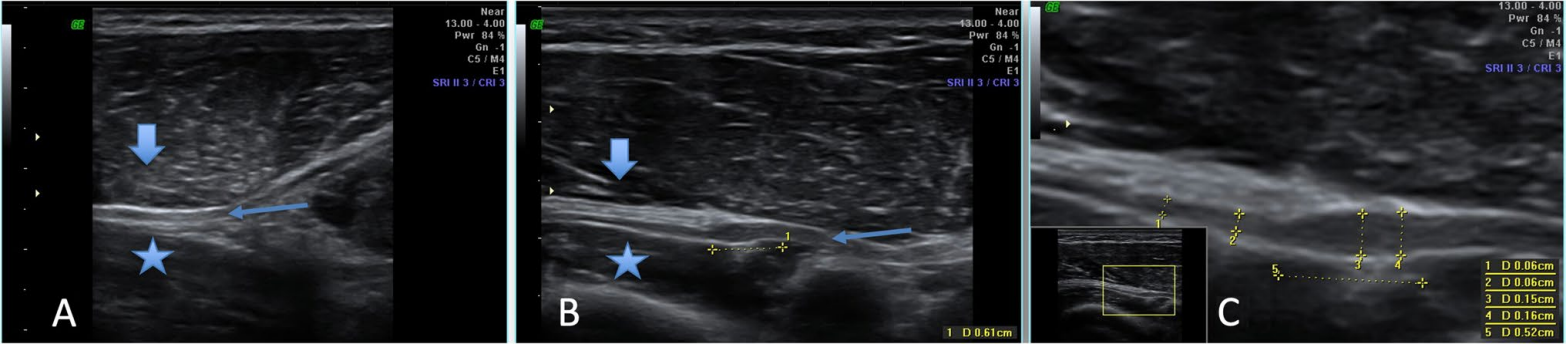
Presents ultrasonographic image of posterior interosseus nerve (PIN). **a** Normal nerve with regular margins and no deformation. Star and arrow marks deep and superficial supinator head respectively. Thin arrow points posterior interosseus nerve. **b** Compression of the nerve by arcade of Frohse with significant broadening of the nerve outline (local oedema formation). Star and arrow marks deep and superficial supinator head respectively. Thin arrow points posterior interosseus nerve. **c** Zoomed view of the changed nerve where comparison of prestenotic nerve edema in comparison with distal part of normal nerve diameter. This is the way of quantification of the nerve enlargement

The mean diameter of the PIN before the supinator canal in symptomatic limbs in patients with PIN syndrome was set to be 3.05 mm (SD 0.68), whilst only 1.85 mm (SD 0.40) in the asymptomatic limbs of the same patients. The mean diameter of the PIN in the supinator canal in symptomatic limbs in patients with PIN syndrome was set to be 3.08 mm (SD 0.59), whilst only 1.77 mm (SD 0.29) in the asymptomatic limbs of the same patients. The PIN was modeled by the arcade of Frohse in a static examination in seven (58.3%) of symptomatic limbs in patients with PIN syndrome. For detailed results of the PIN syndrome examination, regarding all of the assessed parameters, please see Table 2. For US images of examined PIN, please see Fig. 4.

**Table 2:** Results of the occurrence and means of the parameters taken for consideration in evaluation of the posterior interosseous nerve (PIN) syndrome regarding both research and control samples

Category		*N*	Diameter of the PIN before the supinator canal [mm]				Diameter of the PIN in the supinator canal [mm]				Diameter of the PIN after the supinator canal [mm]				Modeling by the arcade of Frohse in a static examination		Modeling by the arcade of Frohse in a dynamic examination		Crossing of the PIN with the recurrent interosseous artery	
			Mean	SD	CI	*p*	Mean	SD		*p*	Mean	SD		*p*	*N* (%)	*p*	*N* (%)	*p*	*N* (%)	*p*
Patients with PIN syndrome	*In symptomatic limbs*	12	3.05	0.68	2.61–3.49	0.00	3.08	0.59	2.70–3.45	0.00	2.90	0.53	2.56–3.24	0.00	7 (58.3%)	0.00	3 (25.0%)	0.06	11 (91.7%)	0.53
	*In asymptomatic limbs*	12	1.85	0.40	1.59–2.11		1.77	0.29	1.58–1.95		1.83	0.31	1.64–2.03		0 (0.0%)		0 (0.0)%		10 (83.3%)	
Patients without PIN syndrome	*In symptomatic limbs*	20	1.68	0.64	1.38–1.97	0.39	1.60	0.69	1.27–1.92	0.13	1.54	0.75	1.19–1.89	0.35	2 (10.0%)	0.15	0 (0.0%)	1.00	9 (45.0%)	0.33
	*In asymptomatic limbs*	20	1.65	0.95	1.20–2.09		1.48	0.71	1.15–1.81		1.51	0.70	1.18–1.83		0 (0.0%)		0 (0.0%)		6 (30.0%)

### Plica syndrome

**Fig. 5:**
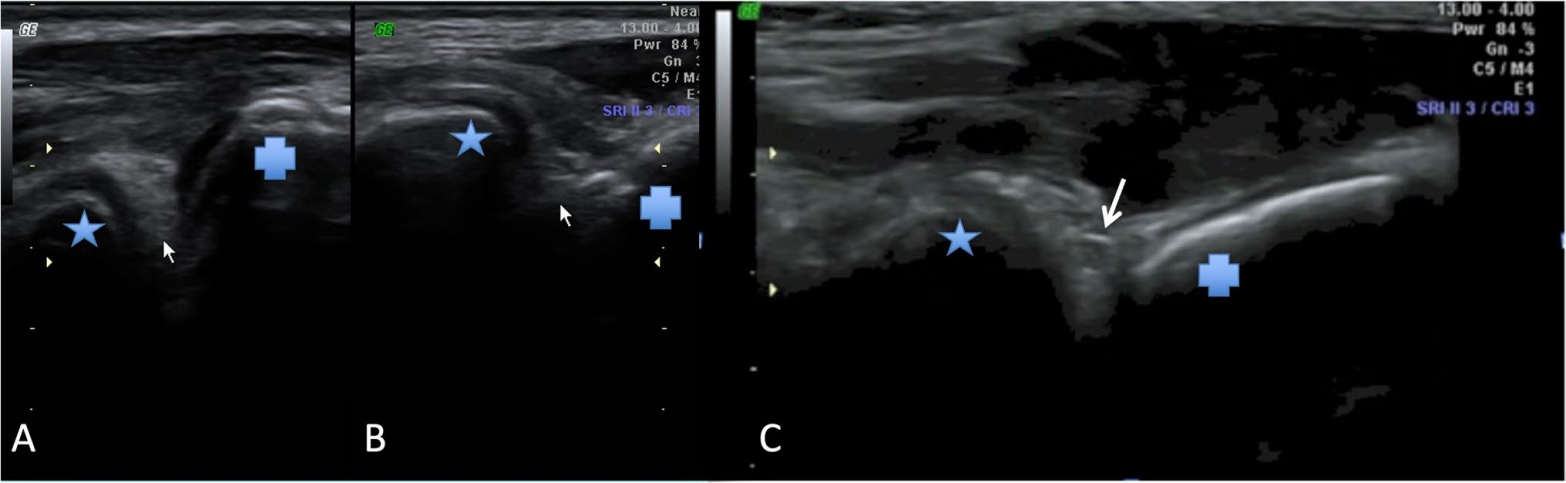
Presents ultrasonographic picture of lateral fold of the elbow. **a** and **b** Fibrotic fold (marked with short arrows) — **a** Fold in relaxed state and **b** compressed fold during flexion what was responsible for clinical symptoms. **c** Swollen fold with formation of accumulation of fluid — a cyst marked with an arrow. Cysts are typically formed in the peripheral areas of the fold where pressure excreted by bone margins are highest. Star presents lateral epicondyle of the humerus, cross marks lateral outline of radial head

The mean plicas’ maximal thickness in symptomatic limbs in patients with plica syndrome was found to be 3.13 mm (SD 1.09). The oedema occurred in 5 (71.4%) of the symptomatic limbs in patients with plica syndrome. The fibrous changes occurred in four (57.1%) symptomatic limbs in patients with plica syndrome. For detailed results of the plica syndrome examination, regarding all of the assessed parameters, please see Table 3. For US images of examined plica, please see Fig. 5.

**Table 3:** Results of the occurrence and means of the parameters taken for consideration in evaluation of the synovial plica of the elbow syndrome regarding both research and control samples

Category		*N*	Plicas’ maximal thickness [mm]				Edema		Fibrous changes		Cystic remodeling		Vascularity in Doppler examination		Modeling by bones	
			Mean	SD	CI	*p*	*N* (%)	*p*	*N* (%)	*p*	*N* (%)	*p*	*N* (%)	*p*	*N* (%)	*p*
Patients with plica syndrome	*In symptomatic limbs*	7	3.13	1.09	2.12–4.14	0.09	5 (71.4%)	0.01	4 (57.1%)	0.02	1 (14.3%)	0.30	3 (42.9%)	0.05	4 (57.1%)	0.02
	*In asymptomatic limbs*	7	2.38	0.98	1.04–2.85		0 (0.0%)		0 (0.0%)		0 (0.0%)		0 (0.0%)		0 (0.0%)	
Patients without plica syndrome	*In symptomatic limbs*	25	2.42	1.34	1.87–2.97	0.16	0 (0.0%)	1.00	1 (4.0%)	0.59	0 (0.0%)	1.00	0 (0.0%)	1.00	0 (0.0%)	1.00
	*In asymptomatic limbs*	25	2.26	1.12	1.80–2.73		0 (0.0%)		0 (0.0%)		0 (0.0%)		0 (0.0%)		0 (0.0%)

### PRTEE and QuickDASH

Both PRTEE and QuickDASH scores were found to statistically significantly correlate with the examined number of sources of the lateral elbow pain. For the detailed results of those correlations, please see Table 4. Additionally, receiver operating characteristic analysis was attempted; however, the results could be potentially biased due to the sample tested; therefore, the authors resigned from reporting those results.

**Table 4:** Correlations between a number of sources of lateral elbow pain and the score obtained in Patient-Related Tennis Elbow Evaluation (PRTEE) questionnaire Quick version of the Disabilities of the Arm (QuickDASH) questionnaire

Category	*N*	*R* Spearman	*t*(N-2)	*p* value
PRTEE	32	0.36	2.13	0.04
QuickDASH	32	0.37	2.21	0.04

## Discussion

The present study evaluates the co-occurrence of the TE, PIN entrapment, and the plica syndrome in light of the complexity of the origin of lateral elbow pain. Our analysis consisted of 31 patients, with a previous diagnosis of chronic TE. Our results show that almost half of the patients (43.7%) did not suffer from only TE. It must be noted that 15.6% of the patients suffered from TE, PIN compression, and plica syndrome simultaneously. Furthermore, TE and plica syndrome was found in 6.3% of the cases, and TE and PIN compression in 18.8%. In light of the present results, the question that must be addressed by the physicians is whether the patient truly has the unsuccessfully treated chronic TE or is simply treated against an inappropriate source of the pain?

Interestingly, the results of the present study show that patients suffering from more than one origin of chronic lateral elbow pain score higher on PRTEE and QuickDASH pain evaluation forms, as the results of both PRTEE and QuickDASH statistically significantly correlated with the number of lateral elbow pain sources. One may postulate that if a patient comes in with chronic lateral elbow pain, and scores exceptionally high on the said evaluation forms, more than one origin of pain may be present. Performing the diagnostic process in a systematic fashion, analogous to what is presented in our study, can increase the probability of diagnosing the patient correctly and subsequently provide appropriate treatment. Our study model and diagnostic approach to chronic lateral elbow pain are easily repeatable, making it a great tool for physicians treating this especially frequent pathology.

All of our patients were already diagnosed with chronic TE prior to our own diagnostic approach. We discovered that oedema, inflammation, tendinosis, and uneven bone attachment area occurred more frequently in patients with TE compared to the asymptomatic limbs (*p* = 0.00). Therefore, the presence of these symptoms may indicate the presence of TE. However, a thorough history inquiry and physical examination are needed for an accurate diagnosis. Other mentionable findings that may help with the diagnosis of TE are signs of abnormal thickening of the tendon and capsule and increased signal intensity within the common extensor origin [14]. The usual treatment for TE is divided into conservative or non-operative and surgical. Non-operative options usually consist of activity changes, physiotherapy, nonsteroidal anti-inflammatory medications, bracing, and acupuncture [15–18]. Furthermore, biotherapy, including autologous blood injections and platelet-rich plasma injections, has been known to have positive effects on patients with TE. Surgical treatment may consist of open approaches, percutaneous techniques, and arthroscopic surgery [14, 15, 19, 20].

The radial nerve bifurcates proximal to the arcade of Frohse to divide into the PIN and the sensory branch of the radial nerve. Arcade of Frohse is also said to be the most frequent compression site of PIN [9, 21]. Studies have shown that a key finding in patients with PIN syndrome, is an enlargement in the dimension of the said nerve. Therefore, this was analyzed in the patients suffering from PIN compression in the present analysis. Our results show that, statistically significantly, the PIN is larger in three different locations when compared to the patients without PIN syndrome; when entering the supinator canal in the supinator canal, and when exiting the supinator canal. Furthermore, the presence of modeling by the arcade of Frohse in a static examination was also proved to be more prevalent in patients with PIN syndrome compared to patients without (*p* = 0.00). Our results further prove that an enlargement of the dimensions of the PIN strongly suggests compression of the said nerve. For refractory cases of PIN syndrome, surgical decompression surgeries are usually performed as the main treatment. These procedures focus on releasing areas of compression of the said nerve. Areas that may be decompressed include releasing fibrous bands non-superficial to the radiocapitellar joint, ligating the leash of Henry (radial recurrent artery), and releasing the arcade of Frohse, amongst others [22].

Plica syndrome was also analyzed in our patient group. The synovial plicae, or the synovial fold, is a common anatomical finding that is reported to occur in 86 to 100% of the cases. However, a symptomatic plicae is said to be much less common [10]. The thickness of the synovial plicae has been heavily discussed, because of its potential significance in the diagnosis of plicae syndrome. Studies in the past have theorized that patients with a synovial fold of > 3.0 mm in thickness (or width, depending on the nomenclature used) may indicate the presence of plica syndrome [23]. However, our results show that, statistically, the synovial plica is not thicker in patients with plica syndrome, compared to patients without, which is consistent with the results established by Bonczar et al. [24]. Interestingly, the presence of oedema, fibrous changes of the fold, increased vascularity detected by Doppler examination, and modeling in bone were statistically higher in patients with plica syndrome than in patients without it (*p* ≤ 0.05). Other significant findings that have been presented in the literature are the occurrence of snapping or clicking during elbow motion, and some local tenderness at the posterolateral aspect of the radiocapitellar joint, and in some cases, in the antero-lateral side [10]. If conservative treatment of a symptomatic synovial fold fails, arthroscopic or open resection of the said fold has been described as both effective and safe.

The present study is not without limitations. This article, to the best of the author's knowledge, is the first in the literature to statistically shed light on the coexistence of these elbow pathologies. Nevertheless, our results are based on 62 elbows and, therefore, should be repeatedly evaluated in further studies by different physicians, ultimately leading to a meta-analysis regarding this issue in the future. Additionally, all evaluated patients were patients from Poland. Therefore, further multinational and multiracial studies should be performed. Furthermore, a receiver operating characteristic analysis regarding the number of sources of lateral elbow pain and the PRTEE and QuickDASH scores should be assessed in further studies with a larger sample. Although the previous study is not without limitations, the authors believe that this study will be the basis for further development of the literature on lateral elbow pain, in effect minimizing the ineffectiveness of its therapy.

## Conclusion

In conclusion, the present article, to the best of the author’s knowledge, is the first in the literature to statistically shed light on the coexistence of these elbow pathologies. The present study demonstrated how a large proportion of patients that were diagnosed with chronic TE, had also additional sources of lateral elbow pain and, because of it did not obtain therapeutic effects of treatment. Our analysis shows how important it is to systematically diagnose patients that present with lateral elbow pain. In order to increase the probability of diagnosing all of the possible aetiologies of the said pain, every patient should go through a thorough physical examination, an ultrasound assessment of the whole elbow region, and fill out pain evaluation forms. The present study showed how the scores of both PRTEE and QuickDASH statistically significantly correlate with the number of lateral elbow pain sources. The clinical characteristics of the three most common causes of chronic lateral elbow pain, meaning TE, PIN compression, and plicae syndrome were also analyzed. Having adequate knowledge about the clinical aspects of these pathologies can help with a more effective differentiation of the etiology of chronic lateral elbow pain and with that, a more efficient and cost-effective treatment plan.

## Data Availability

The data that support the findings of this study are available from the corresponding author, upon reasonable request.

## Declarations

### Disclaimer

The funders had no role in the study’s design, data collection and analysis, decision to publish, or preparation of the manuscript.
